# Gemcabene downregulates inflammatory, lipid-altering and cell-signaling genes in the STAM™ model of NASH

**DOI:** 10.1371/journal.pone.0194568

**Published:** 2018-05-30

**Authors:** Daniela Carmen Oniciu, Taishi Hashiguchi, Yuichiro Shibazaki, Charles L. Bisgaier

**Affiliations:** 1 Gemphire Therapeutics Inc., Livonia, MI, United States of America; 2 SMC Laboratories, Inc., 2-16-1 Minami-Kamata Ota-City, Tokyo, Japan; Medizinische Fakultat der RWTH Aachen, GERMANY

## Abstract

**Background and aims:**

Non-alcoholic fatty liver disease (NAFLD) and non-alcoholic steatohepatitis (NASH) can advance, if untreated, to liver fibrosis, cirrhosis, hepatocellular carcinoma, liver failure and liver-related death. In the United States, NASH affects approximately 2–5% of the population and an additional 10–30% have NAFLD. The number of drugs in development for NASH is growing steadily, along with nonclinical models to support prediction of clinical success. Here we evaluate gemcabene, a first-in-class clinical candidate for dyslipidemia, for its potential utility, based on its combined lipid-lowering and anti-inflammatory efficacy in clinical trials, in a preclinical model of NASH.

**Methods:**

Gemcabene was evaluated in the STAM™ murine model of NASH. Gemcabene intervention in mice made diabetic with streptozotocin and fed a high fat high-caloric diet was assessed for changes in plasma, and hepatic histological and mRNA markers of lipid metabolism and inflammation.

**Results:**

Gemcabene significantly downregulated hepatic mRNA markers of inflammation (TNF-α, MCP-1, MIP-1β, CCR5, CCR2, NF-κB), lipogenesis and lipid modulation (ApoC-III, ACC1, ADH-4, Sulf-2), and fibrosis (TIMP-1 and MMP-2). These effects are important for the prevention of steatosis, inflammation, and hepatocyte ballooning (*i*.*e*., the components of the NAFLD Activity Score or NAS), and inhibition of fibrosis progression, and were observed following treatment with gemcabene.

**Conclusions:**

These non-clinical findings corroborate with existing clinical data to support the clinical evaluation of gemcabene as a potential new treatment for NASH.

## Introduction

Non-alcoholic fatty liver disease (NAFLD) and its severe form non-alcoholic steatohepatitis (NASH), can advance to liver fibrosis, liver cirrhosis, liver failure or hepatocellular carcinoma (HCC) if left untreated [1]. As modern civilization has progressed from an active subsistence to a more sedentary life style, the excess of high-caloric diets and lack of physical activity has dramatically increased the incidence of obesity, metabolic syndrome, diabetes, NAFLD and NASH. Nowadays, even children and adolescents develop early onset liver disease. A systematic review and meta-analysis of children has indicated that NAFLD is present in 7.6% of the general population and in 34.2% of the obese population [2]. The adaptation of human physiology to the current sedentary environmental factors has challenged the medical community to address this emerging health epidemic and evaluate strategies to prevent or regress fatty liver disease.

NAFLD and NASH are often hepatic components of systemic disorders, such as type II diabetes mellitus (T2DM) [3], insulin resistance [4], hyperlipidemia and visceral obesity [5] in direct correlation with the metabolic syndrome (MS) [6] and increasing the risk of cardiovascular diseases [7, 8]. The disease spectrum includes hepatic steatosis, lobular inflammation with hepatocellular ballooning, and liver fibrosis progressing into cirrhosis [4, 9, 10]. There are no approved treatments for NASH. However, although not indicated for NASH, medicines such as pioglitazone and vitamin E have shown significant improvement in hepatic steatosis and inflammation, but not in hepatic fibrosis, nor have they reduced progression to end-stage NAFLD [1, 11]. NASH is on a dramatic rise in various populations on all continents, and can progress, undetected, to severe irreversible conditions that lead to liver-related mortality [12].

**Fig 1:**
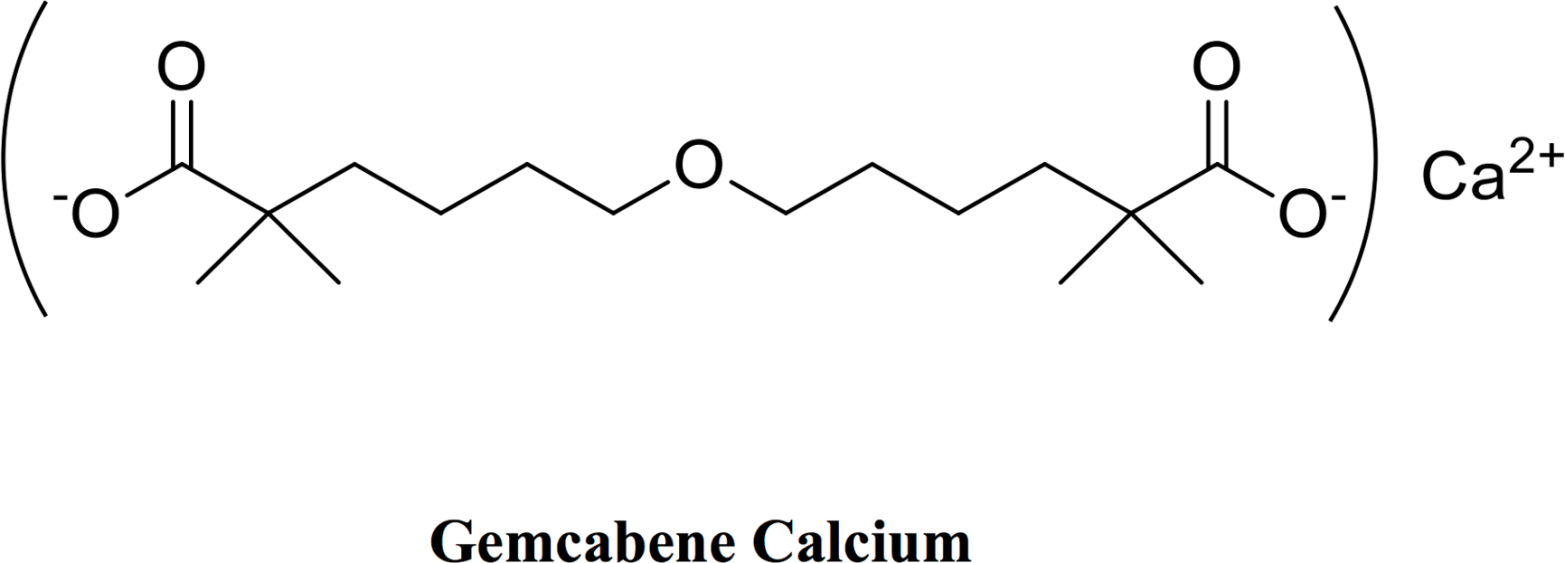
Structure of gemcabene calcium.

Translational medicine in NASH currently merges detection techniques, disease markers and emerging treatments for the identification of potential clinical drug candidates, many of which have already been initially developed for dyslipidemia. The clinical candidate gemcabene calcium (gemcabene) follows the same path. Gemcabene is a “fraudulent” fatty acid discovered by screening a series of compounds with structural features believed to favorably modulate plasma lipids, lipoproteins and apolipoproteins levels (Fig 1) [13, 14]. Many biological effects of gemcabene in preclinical models [13, 15] have been translated to clinical findings in dyslipidemia patients [16, 17]. For example, preclinical studies in chow-fed male Sprague-Dawley rats revealed that gemcabene elevates HDL-C and ApoE, and reduces LDL-C, VLDL-C, ApoB, ApoC-II, ApoC-III and triglycerides (TG) [13]. A comprehensive molecular mechanism by which gemcabene manifests this pharmacological profile is not entirely understood, but it has been shown that it includes reduction of overall hepatic *de novo* triglyceride and cholesterol synthesis, and increased VLDL clearance *via* reduction of hepatic ApoC-III mRNA and plasma ApoC-III levels [18]. Such pleotropic mechanistic features translate to clinical observations: reduction of plasma VLDL-C, LDL-C, and TG [16, 17], and elevation of HDL-C [16]. Preclinical studies showed that gemcabene also reduces CRP and inflammation (*e*.*g*., in the rat arthritis model [15, 19]), which translated to humans [17]). Many of these markers are associated with risk of cardiovascular disease [20, 21], as well as NASH [22].

Recent clinical trials have shown that improvement in NASH or fibrosis are likely dependent on combined hepatic lipid-lowering and anti-inflammatory properties of a drug candidate. Prior research on gemcabene showed that such effects were translated from rodents to humans [13, 15, 16, 17], and therefore a murine model that can span the progression from NAFLD through HCC may be adequate for a proof-of-concept experiment on the effect of gemcabene on NASH disease progression.

No NAFLD model is perfect [23]. For instance, the methionine/choline deficient diet (MCD) fed mouse [24] develops hepatic steatosis and body weight loss without developing insulin resistance. The high-fat/high-calorie diet (HFC)-fed mouse features obesity, hepatic steatosis with mild injury, and insulin resistance [24]. Genetic deficient ob/ob (leptin deficient), db/db mice (leptin-receptor defective) or Zucker rats (leptin-receptor deficient), do not develop steatosis implicitly, unless fed an MCD or an HFC diet [25]. The STAM™ model of NASH-HCC is an HFC-fed mouse model, in which the pathological progression is very similar to that in humans, as these mice develop liver steatosis, inflammation, and partial fibrosis [26, 27]. Therefore, we selected this model for our proof-of-concept experiment to determine whether or not gemcabene could affect the inhibition of NAFLD/NASH progression.

## Materials and methods

Animal housing and care were in compliance with the recommendations of Directive 86/609/EEC, and protocol approvals were obtained from SMC Laboratories IACUC (approval number: S062). All animals were housed and cared for in accordance with the Japanese Pharmacological Society Guidelines for Animal Use.

Effects of gemcabene on NASH parameters were studied in the STAM™ model of NASH-HCC. Information on the preparation of gemcabene, study design, protocol, and study report are presented in the Supplementary Material online (S1).

Briefly, two-day old neonatal C57BL/6 male mice were administered low-dose streptozotocin (STZ), and were subsequently fed a HFC diet from 4 weeks of age. The mice develop liver steatosis and diabetes, reaching steatohepatitis within 3 weeks (*i*.*e*., at about 7 weeks of age), followed by cirrhosis within 8 weeks (i.e., at about 12 weeks of age), and HCC within 16 weeks (*i*.*e*., at about 20 weeks of age) (see Fig A in S1 File) [26, 27]. In the current study, mice were administered daily oral gemcabene starting at week 6 of age and were sacrificed at week 9 of age, a period where the mice are expected to have reached the steatohepatitis phase of the disease and a mild hepatic fibrotic stage [26, 27]. Telmisartan (with antisteatotic, anti-inflammatory and antifibrotic effects in STAM™ mice) was used as a positive comparator [28]. One baseline reference group was administered vehicle at day 2 of age, was chow-fed until sacrifice (week 9 of age), and from week 6 of age was vehicle-treated until sacrifice (week 9 of age). Five STAM™ groups were STZ-treated at day 2 of age and fed a HFC-diet beginning with week 4 of age. These STAM™ groups were orally administered from week 6 of age one of the following: water-vehicle, gemcabene calcium at 30, 100 and 300 mg/kg daily (calculated as gemcabene free acid) in water as a vehicle, or telmisartan (Micardis^®^) 10 mg/kg daily in water as a vehicle. All groups were sacrificed at week 9 of age.

**Fig 2:**
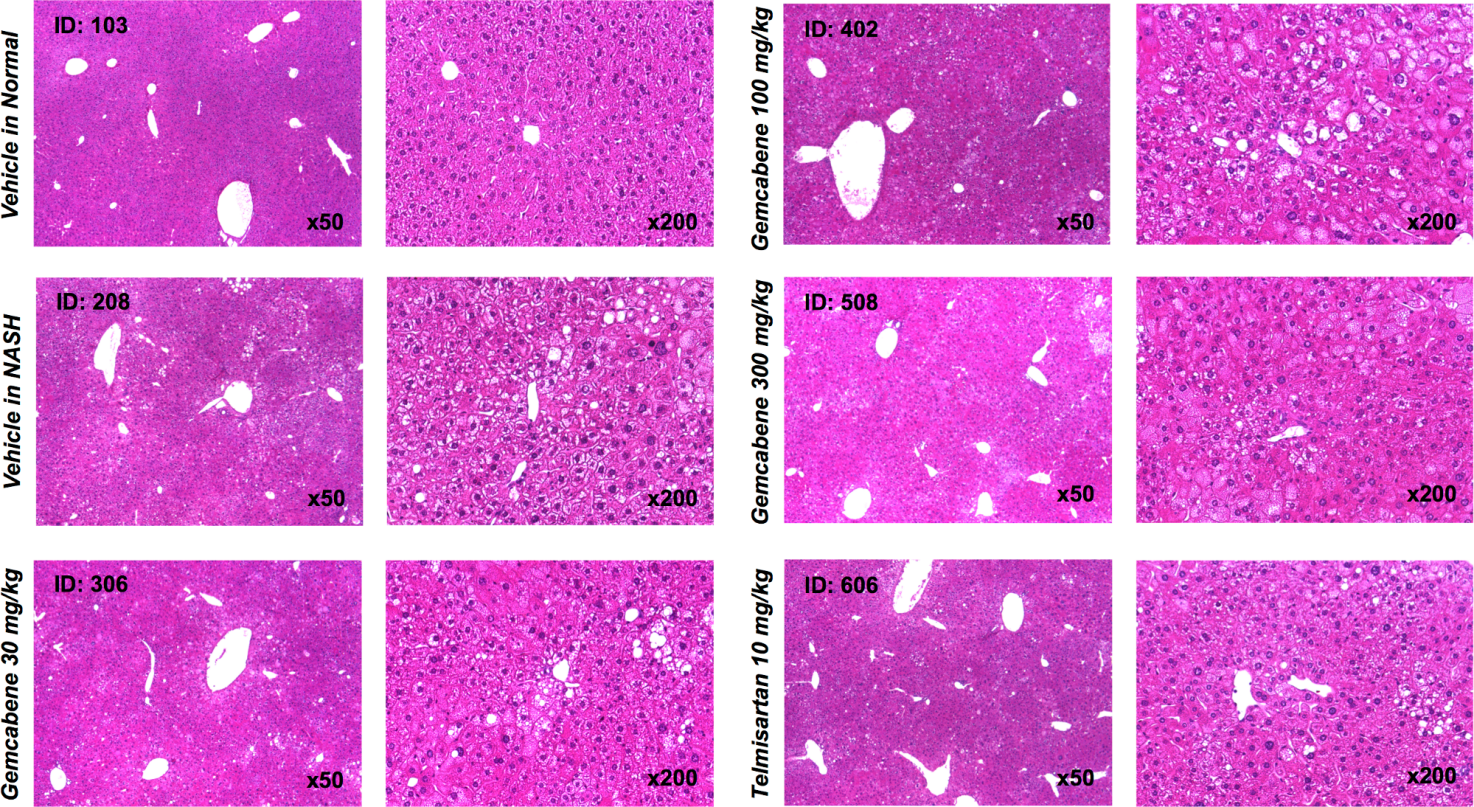
Representative photomicrographs of hematoxylin and eosin-stained liver sections.

**Fig 3:**
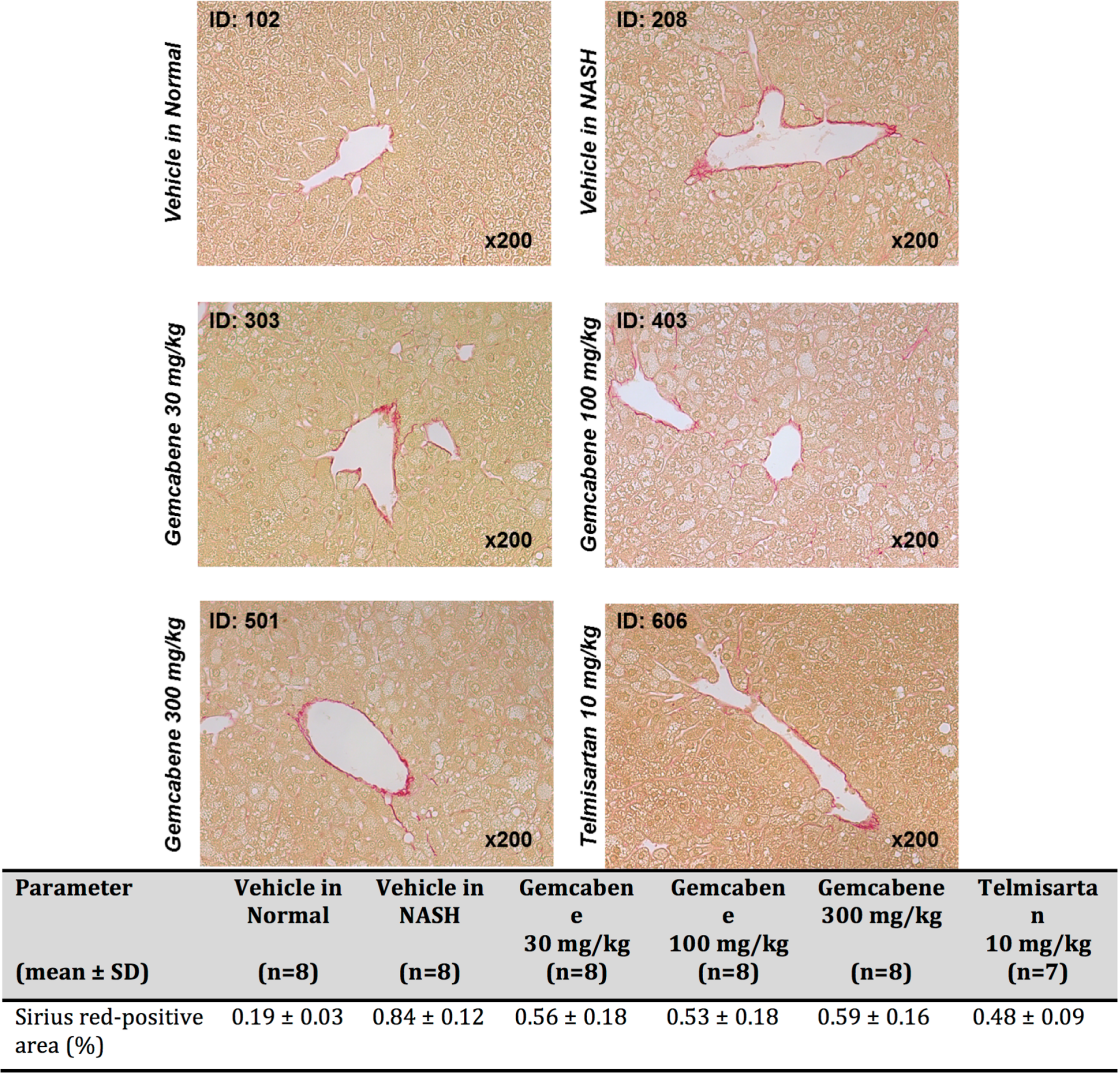
Representative photomicrographs of Sirius red-stained liver sections.

**Fig 4:**
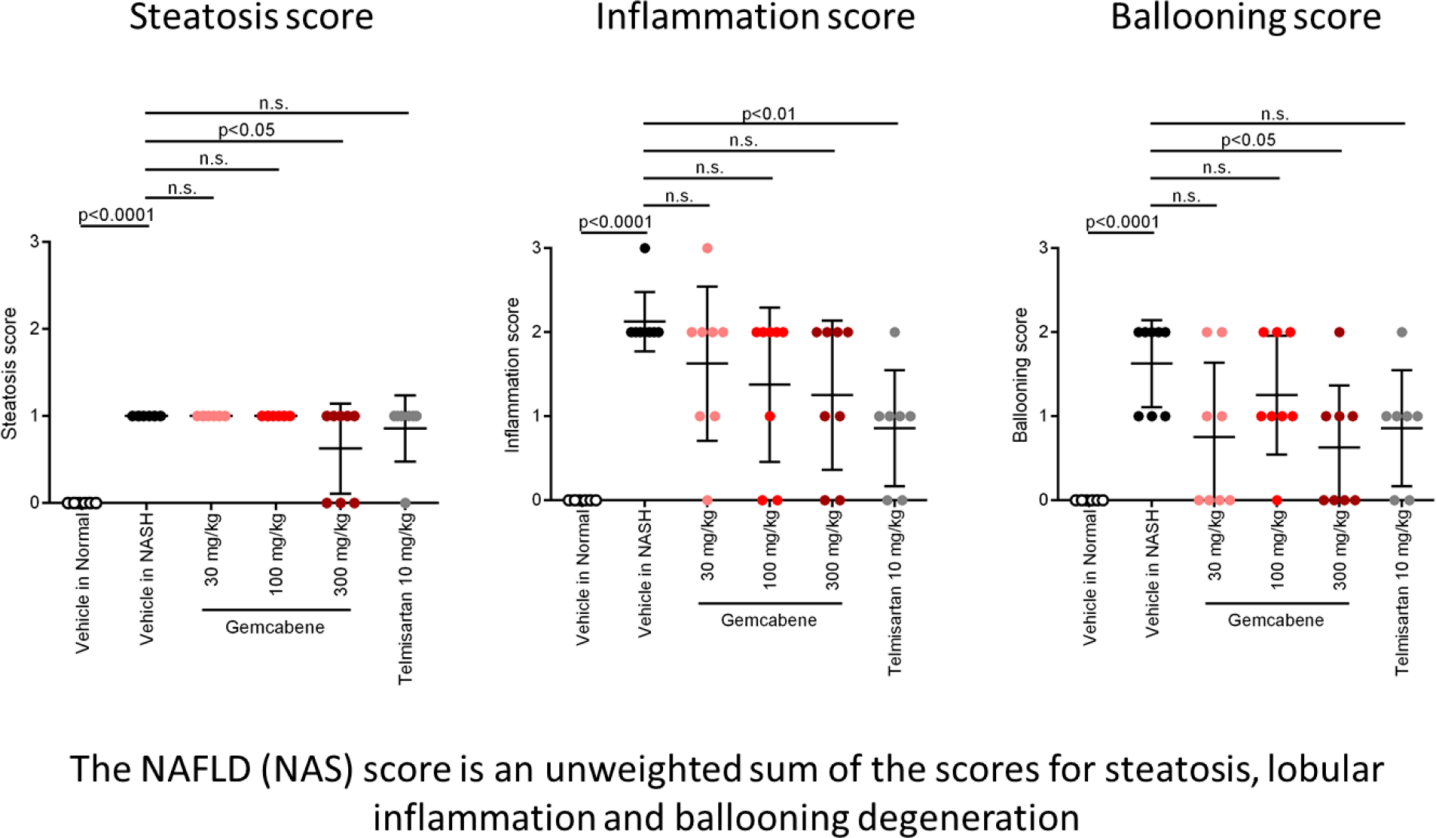
Components of the NAFLD Activity Score (NAS).

**Fig 5:**
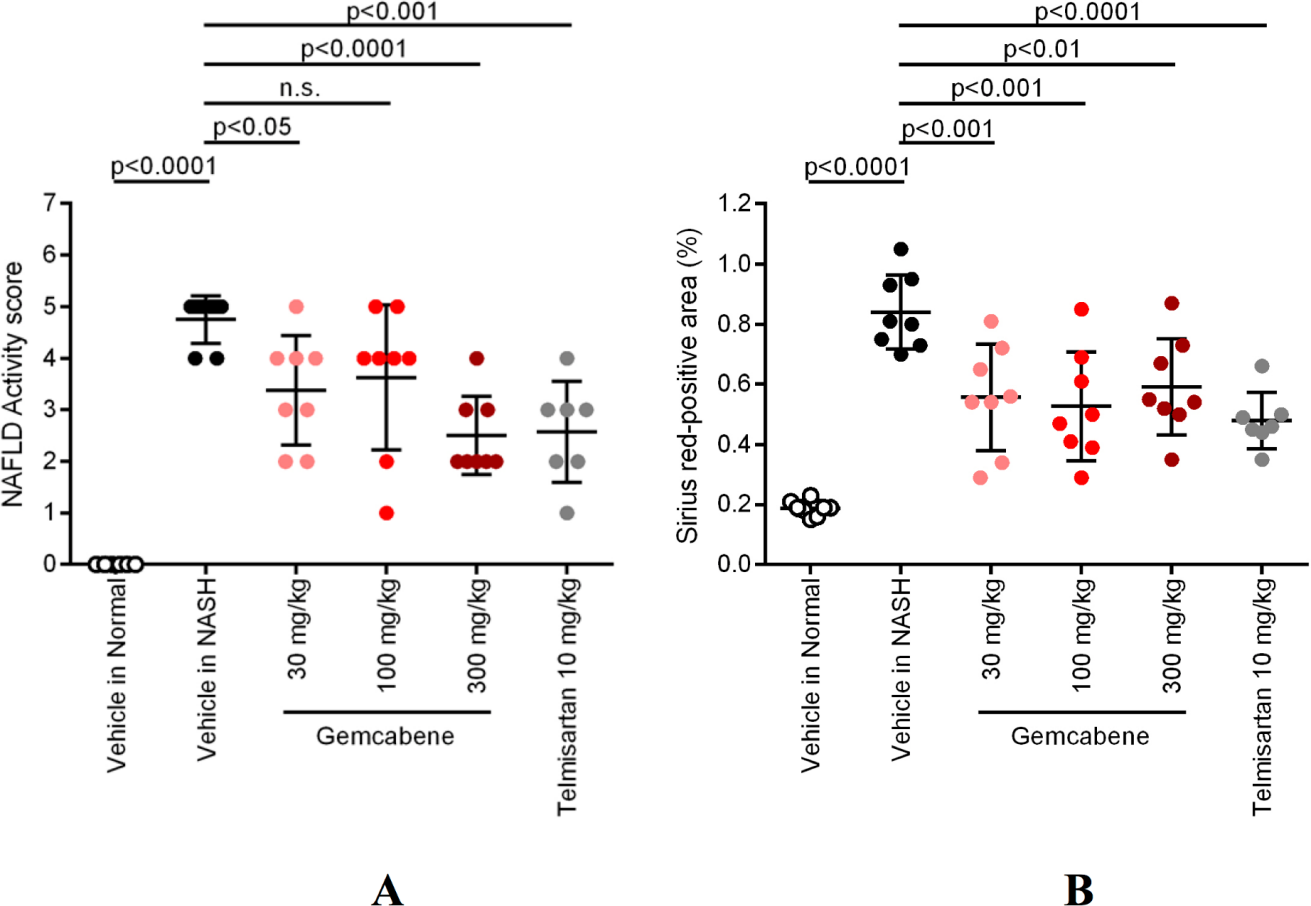
A) NAS Score; B) Fibrosis area.

The biochemistry panel (hepatic lipids, fasting glucose, transaminases and other parameters) is included in Table D in S1 File of the SM. Relevant parameters for gemcabene efficacy related to liver disease are displayed as follows: hepatic pathology (Figs 2 and 3), NAFLD activity score (NAS, a composite of steatosis, lobular inflammation, and hepatocellular ballooning (Table 1, Figs 4 and 5A)), and fibrosis (Fig 5B). All sections were analyzed by a pathologist in a blinded fashion.

**Table 1:** Overview of the NAFLD Activity Score (NAS).

Group	n	Score											NAS mean ± SD (P values)
		Steatosis				Lobular Inflammation				Hepatocyte Ballooning			
		0	1	2	3	0	1	2	3	0	1	2	
Vehicle in Normal	8	8	-	-	-	8	-	-	-	8	-	-	0.0 ± 0.0
Vehicle in NASH	8	-	8	-	-	-	-	7	1	-	3	5	4.8 ± 0.5 (p<0.0001)^a^
Gemcabene 30 mg/kg	8	-	8	-	-	1	2	4	1	4	2	2	3.4 ± 1.1 (p<0.05)^b^
Gemcabene 100 mg/kg	8	-	8	-	-	2	1	5	-	1	4	3	3.6 ± 1.4 (ns)^b^
Gemcabene 300 mg/kg	8	3	5	-	-	2	2	4	-	4	3	1	2.5 ± 0.8 (p<0.0001)^b^
Telmisartan 10 mg/kg	7	1	6	-	-	2	4	1	-	2	4	1	2.6 ± 1.0 (p<0.001)^b^
**Item**		**Score**				**Extent**							
Steatosis		0				<5%							
		1				5–33%							
		2				>33–66%							
		3				>66%							
Lobular Inflammation		0				No foci							
		1				<2 foci/200x							
		2				2–4 foci/200x							
		3				>4 foci/200x							
Hepatocyte Ballooning		0				None							
		1				Few balloon cells							
		2				Many cells/prominent ballooning							
		**Vehicle in NASH**				**Gemcabene** **30 mg/kg**		**Gemcabene** **100 mg/kg**		**Gemcabene** **300 mg/kg**			**Telmisartan** **10 mg/kg**
		**(vs. Vehicle in Normal)**				**(vs. Vehicle in NASH)**							
**Histological** **analyses**	**NAFLD Activity score**	▲(p<0.0001)				▼ (p<0.05)		(NS)		▼(p<0.0001)			▼(p<0.001)
	**Steatosis score**	▲ (p<0.0001)				(NS)		(NS)		▼(p<0.05)			(NS)
	**Inflammation score**	▲ (p<0.0001)				(NS)		(NS)		(NS)			▼(p<0.01)
	**Ballooning score**	▲ (p<0.0001)				(NS)		(NS)		▼(p<0.05)			(NS)
	**Fibrosis area**	▲ (p<0.0001)				▼(p<0.001)		▼(p<0.001)		▼(p<0.01)			▼ (p<0.0001)

- no significant difference

▲ significant increase

▼ significant decrease

^a^ Compared to Vehicle Normal

^b^ Compared to Vehicle NASH

NAS was calculated using the method described by Kleiner et al. [10] (see also S1 File online). NAS is defined as the sum of the histological scores for steatosis, lobular inflammation and hepatocyte ballooning. Each score was calculated as displayed in Table 1.

Various gene expression markers of liver metabolism were evaluated by Real-Time PCR (RT-PCR) in all groups (Table 2). In order to calculate the relative mRNA expression level, the expression of each gene was normalized to that of the reference gene 36B4 (gene symbol: Rplp0). Information on the PCR-primer sets and the plate layout is presented in Table Ab in S1 File. Results are normalized with values for the vehicle-treated normal group.

**Table 2:** Gene expression analysis.

Parameter	Vehicle in Normal	^a^Vehicle in NASH	^b^Gemcabene 30 mg/kg	^b^Gemcabene 100 mg/kg	^b^Gemcabene 300 mg/kg	^b^Telmisartan 10 mg/kg	Function
(mean ± SD)	(n = 8)	(n = 8)	(n = 8)	(n = 8)	(n = 8)	(n = 7)	
TNF-α	1.0 ± 0.3	3.6 ± 1.0 (p<0.0001)	4.0 ± 1.8 (NS)	2.0 ± 0.8 (p<0.05)	1.9 ± 0.7 (p<0.05)	3.0 ± 1.2 (NS)	Inflammation– ↑ in NAFLD
NF-κB	1.0 ± 0.1	1.3 ± 0.2 (p<0.001)	1.3 ± 0.2 (NS)	0.9 ± 0.1 (p<0.0001)	0.8 ± 0.1 (p<0.0001)	1.1 ± 0.1(p<0.05)	↑ Proinflammatory genes (cytokines, chemokines, and adhesion molecules)
CRP	1.0 ± 0.2	1.0 ± 0.2 (NS)	0.9 ± 0.2 (NS)	0.6 ± 0.1 (p<0.0001)	0.5 ± 0.1 (p<0.0001)	0.9 ± 0.1 (p<0.0001)	Surrogate marker of hepatic inflammation in NASH
MCP-1	1.0 ± 0.4	3.6 ± 1.7 (p<0.001)	3.2 ± 1.5 (NS)	1.7 ± 0.7 (p<0.01)	1.6 ± 0.7 (p<0.01)	2.1 ± 1.0 (p<0.05)	↑ Chemokine following inflammation in stellate cells NAFLD
α-SMA	1.0 ± 0.3	3.1 ± 0.9 (p<0.0001)	2.6 ± 0.6 (NS)	2.4 ± 0.9 (NS)	2.5 ± 0.7 (NS)	2.3 ± 0.7 (NS)	↑TNF-α ↑expression and deposition of α-SMA
MMP-2	1.0 ± 0.2	1.9 ± 0.7 (p<0.01)	1.7 ± 0.5 (NS)	0.5 ± 0.2 (p<0.0001)	0.9 ± 0.2 (p<0.001)	1.4 ± 0.7 (NS)	Degrades type-IV collagen; involved in NAFLD pathogenesis
TIMP-1	1.0 ± 0.3	12.9 ± 9.0 (p<0.0001)	9.9 ± 4.9 (NS)	3.8 ± 1.6 (p<0.01)	4.4 ± 2.1(p<0.01)	8.6 ± 5.1 (NS)	↓ Collagenase activity
MIP-1β	1.0 ± 0.2	5.6 ± 2.0 (p<0.0001)	5.4 ± 3.2 (NS)	2.3 ± 0.9 (p<0.01)	2.8 ± 1.4 (p<0.05)	3.9 ± 1.5 (NS)	↑ Pyrogenic, mitogenic, induce the synthesis and release of pro-inflammatory cytokines such as IL-1, IL-6 and TNF-α from fibroblasts and macrophages
CCR5	1.0 ± 0.2	2.3 ± 0.7 (p<0.0001)	2.4 ± 0.9 (NS)	1.4 ± 0.3 (p<0.01)	1.3 ± 0.3 (p<0.01)	1.5 ± 0.3 (p<0.05)	Progression of hepatic inflammation and fibrosis
CCR2	1.0 ± 0.2	3.5 ± 1.7 (p<0.0001)	3.3 ± 1.0 (NS)	1.6 ± 0.4 (p<0.001)	1.7 ± 0.7 (p<0.01)	2.4 ± 0.8 (NS)	Monocyte/macrophage recruitment and tissue infiltration, hepatic stellate cell activation
ACC1	1.0 ± 0.2	0.9 ± 0.2 (NS)	1.0 ± 0.1 (NS)	0.7 ± 0.1 (p<0.05)	0.8 ± 0.1 (NS)	0.7 ± 0.1 (p<0.01)	Hepatic lipogenesis (CoA synthesis, FFA synthesis and oxidation)
ACC2	1.0 ± 0.2	0.5 ± 0.1 (p<0.0001)	0.6 ± 0.2 (NS)	0.4 ± 0.1 (NS)	0.5 ± 0.1 (NS)	0.3 ± 0.1 (p<0.05)	
ApoC-III	1.0 ± 0.2	0.7 ± 0.1 (p<0.001)	0.7 ± 0.1 (NS)	0.5 ± 0.0 (p<0.01)	0.4 ± 0.1 (p<0.0001)	0.8 ± 0.2 (NS)	Clearance of triglyceride-rich lipoproteins
SREBP-1	1.0 ± 0.3	0.9 ± 0.2 (NS)	0.9 ± 0.2 (NS)	0.9 ± 0.2 (NS)	0.7 ± 0.1 (NS)	0.7 ± 0.2 (NS)	Regulates genes required for lipogenesis (e.g. LDL gene) and glucose metabolism; ↑ de novo C synthesis and uptake and FA synthesis
Sulf-2	1.0 ± 0.3	5.2 ± 1.2 (p<0.001)	5.1 ± 1.1 (NS)	3.8 ± 0.7 (p<0.05)	3.3 ± 0.9 (p<0.001)	3.9 ± 0.9 (NS)	Sulfation of heparan sulfate proteoglycans (HSPGs), (i.e., Syndecan-1), in the extracellular hepatic matrix, critical signaling pathway
PNPLA3	1.0 ± 0.4	0.3 ± 0.1 (p<0.0001)	0.3 ± 0.1 (NS)	0.2 ± 0.1 (NS)	0.2 ± 0.2 (NS)	0.1 ± 0.0 (NS)	Isomorphs associated with insulin resistance and NASH
ADH-4	1.0 ± 0.2	0.9 ± 0.3 (NS)	0.8 ± 0.2 (NS)	0.6 ± 0.1 (p<0.05)	0.5 ± 0.1 (p<0.001)	0.6 ± 0.2 (p<0.01)	Oxidation of ethanol to aldehydes and ketones; reduces NAD to NADH
LDL receptor	1.0 ± 0.1	0.9 ± 0.2 (NS)	0.9 ± 0.2 (NS)	0.9 ± 0.2 (NS)	0.8 ± 0.1 (NS)	0.7 ± 0.3 (NS)	C homeostasis, cell signaling

^a^ Compared to Vehicle Normal

^b^ Compared to Vehicle NASH

Abbreviations: ACC = Acetyl-CoA carboxylase; ADH = Alcohol dehydrogenase; C = cholesterol; CCR = C-C chemokine receptor; CRP = C-reactive protein; FA = Fatty acid; FFA = free fatty acid; HSPGs = heparan sulfate proteoglycans; LDL = low-density lipoprotein; MCoA = Malonyl-CoA; MCP = Monocyte chemotactic protein; MMP = Matrix metalloproteinase; MIP = Macrophage inflammatory protein; NAD = nicotinamide adenine dinucleotide; NF-κB = Nuclear factor-kappa B; PNPLA = Patatin-like phospholipase-containing domain; SMA = Smooth muscle actin; SPF = Specific pathogen-free; SREBP = Sterol regulatory element-binding protein; Sulf = Sulfatase; TIMP = Tissue inhibitor of metalloproteinase; TNF = Tumor necrosis factor.

## Results

### Gemcabene diminishes micro- and macro-vesicular fat deposition, hepatocellular ballooning and inflammatory cell infiltration

Representative photomicrographs of hematoxylin and eosin (H&E)-stained liver sections are presented in Fig 2. H&E-stained liver sections from the vehicle-treated NASH mice exhibited micro- and macro-vesicular fat deposition, hepatocellular ballooning (degeneration of hepatocytes) and inflammatory cell infiltration compared to the vehicle-treated normal mice. Gemcabene-treated (300 mg/kg) and telmisartan-treated mice showed less steatosis than the vehicle-treated NASH mice.

Gemcabene-treated 30 and 300 mg/kg groups and telmisartan-treated mice showed less lobular inflammation and ballooning than the vehicle-treated NASH mice (Fig 2, Table 1), and showed significant reduction in the NAS (Fig 5A) compared with the vehicle-treated NASH mice. Although trending lower, there was no significant difference in NAS between the gemcabene–treated (100 mg/kg) and the vehicle-treated NASH mice.

**Fig 6:**
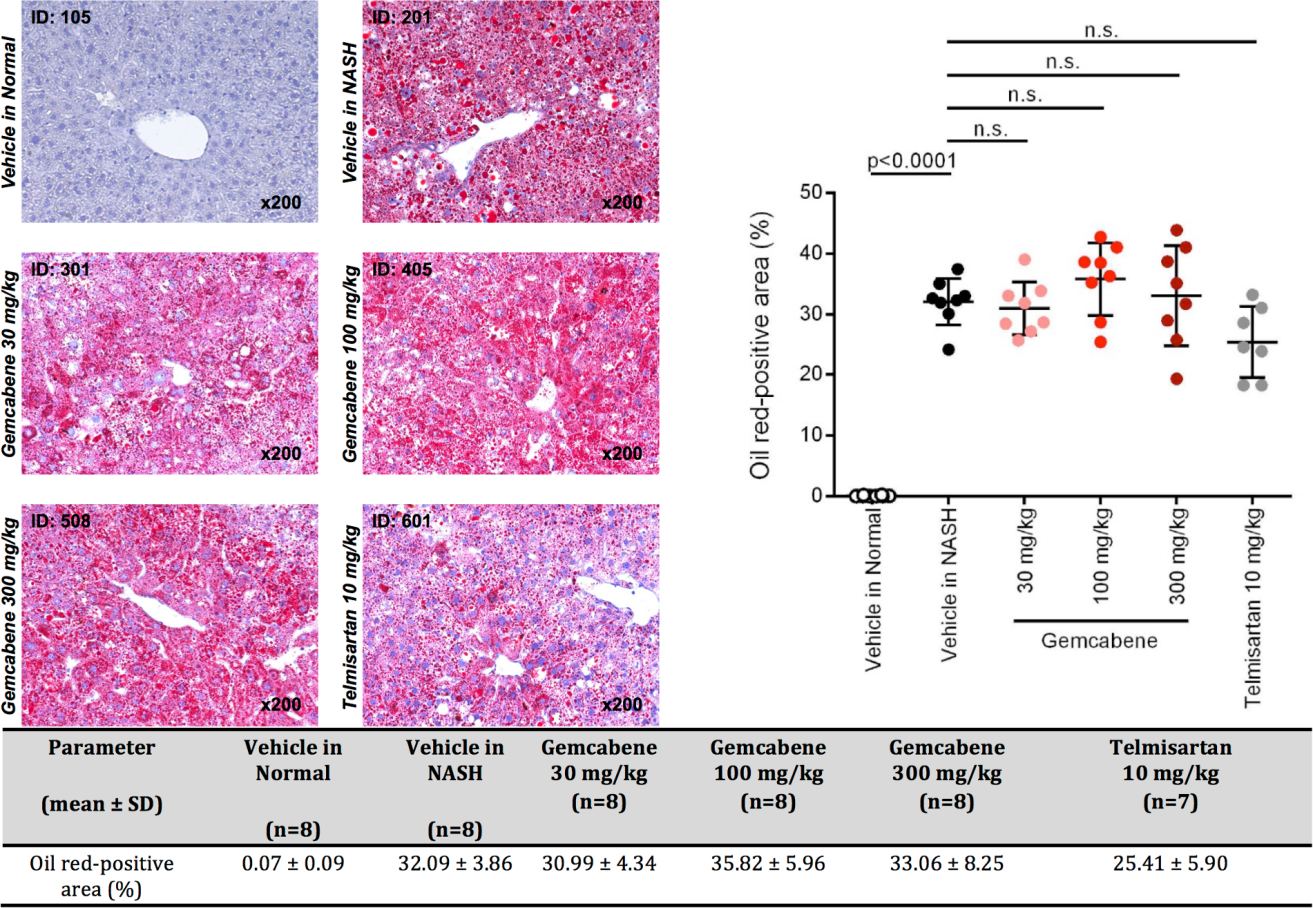
Oil Red-O Staining Analysis: A—Representative photomicrographs of Oil Red Stained Liver Sections; B—Fat Deposition Area.

Representative information on Oil Red-O staining is shown in Fig 6. Liver sections from the Vehicle in the NASH group exhibited micro- and macro-vesicular fat deposition in the hepatocytes compared with the Vehicle in the Normal group, with a significant increase in the fat deposition area (Oil Red-positive area) compared with the Vehicle in Normal group. There were no significant differences in the fat deposition area between the Vehicle in NASH group and any of the treatment groups. This finding is discordant with that of the steatosis score, and therefore should be interpreted cautionarily. Indeed, the quantification of Oil Red-O staining of sections containing extremely small fat droplets, which are smaller than microvesicular fat droplets, are counted as a positive signal. However, when calculating steatosis in terms of reduction of steatosis score in NAS, only micro- and macrovesicular fat droplets are taken into account. In the calculation of steatosis in our experiment, an acceptable score was obtained for gemcabene-treated mice at the 300 mg/kg daily dose. We consider this discrepancy to be the reason why the results of the steatosis score and the Oil Red-O staining do not corroborate with each other.

### Gemcabene reduced the fibrosis area

Sirius Red stained liver sections (Fig 3) from the vehicle-treated NASH mice showed increased collagen deposition in the hepatic lobule pericentral region compared with the vehicle-treated normal mice. All gemcabene and telmisartan-treated groups showed significant decreases in fibrosis area compared to the vehicle-treated NASH mice (Fig 5B).

There were no significant differences in liver hydroxyproline contents between the vehicle-treated NASH mice and any of the treatment groups (gemcabene- or telmisartan-treated, see Table C in S1 File). Gemcabene did not show a reduction in the hydroxyproline levels, most likely due to the lack of efficiency of such measurements when the fibrosis stage is mild.

### Gene expression analysis show downregulation of inflammatory, fibrosis and cell signaling genes

Gemcabene modulates the mRNA expression of a multitude of hepatic genes that were shown to play a significant role in liver homeostasis and injury [29]. Table 2 presents the results of the gene expression RT-PCR measurements normalized to the non-treated group.

Gemcabene-treated 100 and 300 mg/kg groups significantly suppressed TNF-α mRNA expression (2.0 ± 0.8 and 1.9 ± 0.7, respectively), while the vehicle-treated NASH mice showed a significant up-regulation in TNF-α mRNA levels (3.6 ± 1.0) compared to the vehicle-treated normal mice. There were no significant differences in TNF-α mRNA levels between the vehicle-treated NASH mice and any other treatment groups.

Similarly, NF-kB mRNA levels are slightly up-regulated in vehicle-treated NASH mice (1.3 ± 0.2) compared to vehicle-treated normal mice. Gemcabene 100 and 300 mg/kg down-regulated NF-kB mRNA expression levels (0.9 ± 0.1 and 0.8 ± 0.1, respectively) compared to the vehicle-treated NASH mice.

Gemcabene 100 and 300 mg/kg treated groups showed a significant reduction in CRP mRNA levels (0.6 ± 0.1 and 0.5 ± 0.1, respectively) compared with the vehicle-treated NASH group (1.0 ± 0.2), consistent with the observed clinical reduction of plasma CRP with gemcabene [17]. No significant differences in CRP mRNA levels were observed for other treatment groups, particularly telmisartan.

The monocyte chemoattractant protein-1 mRNA (MCP-1, also called chemokine (C-C motif) ligand 2 or CCL2) in the vehicle-treated NASH mice was significantly up-regulated compared with the vehicle-treated normal mice. Gemcabene 100 and 300 mg/kg treated mice significantly down-regulated MCP-1 mRNA expression levels compared with the vehicle-treated NASH mice (1.7 ± 0.7 and 1.6 ± 0.7, respectively, versus 3.6 ± 1.7), and higher than telmisartan (2.1 ± 1.0).

Chemokine (C-C motif) ligand 4 (CCL4), also known as macrophage inflammatory protein-1β (MIP-1β) is an inflammatory marker known to be elevated in NAFLD. Hepatic MIP-1β mRNA levels were significantly higher in the vehicle-treated NASH mice (5.6 ± 2.0) compared with vehicle-treated normal mice. Gemcabene 100 and 300 mg/kg treated mice and telmisartan, showed significant down-regulation of MIP-1β mRNA levels (2.3 ± 0.9, 2.8 ± 1.4, and 3.9 ± 1.5, respectively).

Expression of MMP-2 and TIMP-1, genes involved in collagen degradation and collagenase activity, respectively, showed similar significant down-regulation patterns in 100 and 300 mg/kg gemcabene -treated mice. Matrix metalloproteinase-2 (MMP-2) mRNA levels are up-regulated in vehicle-treated NASH mice (1.9 ± 0.7), while gemcabene-treated mice at 100 and 300 mg/kg doses significantly down-regulated the MMP-2 mRNA expression levels (0.5 ± 0.2 and 0.9 ± 0.2, respectively).

Tissue inhibitor of metalloproteinase 1 (TIMP-1) mRNA levels were significantly up-regulated in the vehicle-treated NASH mice (12.9 ± 9.0) compared to the vehicle-treated normal mice. Gemcabene 100 and 300 mg/kg treated groups significantly down-regulated TIMP-1 mRNA expression (3.8 ± 1.6 and 4.4 ± 2.1, respectively).

### Gemcabene’s impact on the CCR2 and CCR5 mRNA expression

Interactions between C-C chemokine receptor types 2 (CCR2) and its ligand, CCL2, mediate fibrogenesis by promoting monocyte/macrophage recruitment and tissue infiltration, as well as hepatic stellate cell activation [30]. Vehicle-treated NASH mice showed significant up-regulation in CCR2 mRNA expression levels (3.5 ± 1.7) compared with the vehicle-treated normal mice. Gemcabene 100 and 300 mg/kg treated groups showed significant down-regulation in CCR2 mRNA expression levels (1.6 ± 0.4 and 1.7 ± 0.7, respectively) and to a greater extent when compared to telmisartan (2.4 ± 0.8).

The chemokine CCL5/RANTES and its receptor, CCR5, play important roles in the progression of hepatic inflammation and fibrosis [30]. Given the role of CCR5 in the NAFLD pathogenesis, we expected CCR5 to be elevated in the vehicle-treated NASH. Indeed, the vehicle-treated NASH group showed a significant increase in CCR5 mRNA levels (2.3 ± 0.). Gemcabene 100 and 300 mg/kg and telmisartan-treated groups significantly down-regulated CCR5 mRNA expression levels (1.4 ± 0.3, 1.3 ± 0.3, and 1.5 ± 0.3, respectively).

### Gemcabene’s effect on genes of lipogenesis and lipid metabolism: ACC-1, ApoC-III, Sulf-2 and SREBP-1 mRNA regulation

Both acetyl-coA carboxylases 1 and 2 (ACC-1 and ACC-2) catalyze the synthesis of malonyl-CoA, the substrate for fatty acid synthesis and the regulator of fatty acid oxidation, major players in the NAFLD pathogenesis [31]. Gemcabene 100 mg/kg and telmisartan-treated mice, down-regulated ACC-1 mRNA expression levels compared to the vehicle-treated NASH mice (0.7 ± 0.1 compared to 0.9 ± 0.2), but had no effect on ACC-2. Similarly, ApoC-III gene expression levels for gemcabene 100 mg/kg and 300 mg/kg were significantly down-regulated compared to the vehicle-treated NASH mice (0.5 ± 0.0 (p<0.01) and 0.4 ± 0.1 (p<0.0001), respectively, compared to 0.7 ± 0.1 (p<0.001)).

Heparan sulfate glucosamine-6-O-endosulfatase-2 (Sulf-2) is one of the sulfatases that modulates the sulfation status of heparan sulfate proteoglycans (HSPGs), particularly Syndecan-1, in the extracellular hepatic matrix, and it also regulates a number of critical signaling pathways involved in diabetes and lipid trafficking [32]. In the current study, vehicle-treated NASH mice showed a significant up-regulation of Sulf-2 mRNA levels compared with the vehicle-treated normal group (5.2 ± 1.2). Gemcabene 100 and 300 mg/kg-treated mice significantly down-regulated Sulf-2 mRNA expression levels (3.8 ± 0.7 and 3.3 ± 0.9, respectively).

The SREBP-1 gene is associated with lipogenesis and regulation of the LDL receptor gene, and its levels are indirectly regulated by cholesterol, insulin and other endogenous molecules [33]. In this experiment, there are no differences in the SREBP-1 mRNA levels between the vehicle-treated NASH mice and any other treatment groups. Similarly, gemcabene and telmisartan showed no effect on the LDL receptor gene expression.

### Regulation of the human alcohol dehydrogenase 4 (ADH-4) gene

ADH-4, associated with NAFLD, contributes to ethanol metabolism at moderate and high concentrations [34]. Induction of NASH in the STAM™ mice had no significant effect on ADH-4 mRNA levels (vehicle-treated NASH and vehicle-treated normal groups have similar values). However, gemcabene 100 and 300 mg/kg and telmisartan-treated groups down-regulated ADH-4 mRNA expression levels compared to the vehicle-treated NASH group (0.6 ± 0.1, 0.5 ± 0.1 and 0.6 ± 0.2, respectively, compared to 0.9 ± 0.3).

## Discussion

Both hepatic lipid flux (*via* fatty acid/triglyceride synthesis [35] or fatty acid delivery [36]), and hepatic inflammation [37, 38] contribute to the NAFLD/NASH progression. Treatments that target one or more metabolic systems to prevent hepatic triglyceride accumulation and reduction of hepatic inflammation may prevent and possibly regress pre-existing NAFLD/NASH.

A comparative evaluation of nonclinical and clinical findings for drugs in development for NASH is displayed in Table E in S1 File. The outcome of NASH trials is forthcoming and provides direction for future targets, but most drugs focused on a single lipid or inflammatory target, *e*.*g*., ACC1 [31], PPARs and FXR (examples in Table E in S1 File), CCR2 [30] and CCR5 [30], or reduction in a chemotaxis protein, such as MCP-1 [30]. Clinical endpoints revolve mostly around reduction in transaminases, resolution of NASH with no worsening in fibrosis, or improvement in fibrosis with no worsening in NASH (Table E in S1 File and, *e*.*g*., https://clinicaltrials.gov/ct2/show/NCT02548351). The NAS composite score is a multi-faceted representation of liver histopathology. As NASH is a heterogeneous disease, patient responses to specific or targeted therapies may differ or would be expected to differ. In the future, individual variability may be overcome by combinations of two or more drugs, which may simultaneously modulate multiple lipogenic, lipid metabolic, and inflammatory targets.

We used the preclinical STAM™ model to evaluate how gemcabene affects several parameters thought to be relevant in the NASH pathophysiology based on prior findings with successful clinical candidates. Medicinal candidates in development for NASH presented in Table E in S1 File (*e*.*g*., pioglitazone, vitamin E, obeticholic acid (OCA), cenicriviroc, linagliptin, pegylated Fibroblast Growth Factor 21 (FGF21)) show effects that suggest the possibility of translatable markers and outcomes from the STAM™ model that could be of potential clinical relevance. Gemcabene in the STAM™ model showed a robust reduction in steatosis and NAS, in similar ranges of magnitude to other drugs in development (Table E in S1 File). Although histologic effects on fibrosis (i.e., collagen deposition around the portal vein) were mild, treatment with 100 and 300 mg/kg gemcabene reduced the mRNA expression levels of TIMP-1, a marker of collagenase activity, similar to the findings with cenicriviroc. In the same line, the mRNA expression level of the MMP-2 gene, controlling the degradation of type-IV collagen and involved in NAFLD pathogenesis, is significantly downregulated by treatment with 100 and 300 mg/kg gemcabene.

Arguably, reduction in steatosis is not solely causal, and drug candidates may also need to have anti-inflammatory activity to be effective. Disease improvement by a few drug candidates currently in development is attributed to their anti-inflammatory properties, which were predicted by gene expression analysis in the STAM™ model (Table E in S1 File). These drugs have been shown to downregulate mRNA expression levels of one or multiple inflammatory genes among TNF-α, MCP-1, MIP-1β, CCR5, CCR2, and NF-κB. Notably, in our study gemcabene was observed to suppress all the inflammatory markers mentioned above (Table 3). This effect is consistent with our earlier findings of reduction in plasma CRP levels seen in clinical trials in patients with dyslipidemia [17] and inhibition of CRP secretion in cell-based assays [15].

**Table 3:** Gene expression and histology summary.

		Gemcabene				
		Vehicle in NASH	Gemcabene 30 mg/kg	Gemcabene 100 mg/kg	Gemcabene 300 mg/kg	Telmisartan 10 mg/kg
		*(vs Vehicle in Normal)*	*(vs Vehicle in NASH)*			
**Gene Expression Analyses**	**TNF-α**	▲ (p<0.0001)	NS	▼ (p<0.05)	▼ (p<0.05)	NS
	**MCP-1**	▲ (p<0.001)	NS	▼ (p<0.01)	▼ (p<0.01)	▼ (p<0.05)
	**α-SMA**	▲ (p<0.0001)	NS	NS	NS	NS
	**TIMP-1**	(p<0.0001)	NS	▼(p<0.01)	▼(p<0.01)	NS
	**SREBP-1**	NS	NS	NS	NS	NS
	**MIP-1β**	▲ (p<0.0001)	NS	▼(p<0.01)	▼(p<0.05)	NS
	**CCR5**	▲ (p<0.0001)	NS	▼(p<0.01)	▼(p<0.01)	▼(p<0.05)
	**CCR2**	▲ (p<0.0001)	NS	▼(p<0.001)	▼(p<0.01)	NS
	**NF-κB**	▲ (p<0.001))	NS	▼(p<0.0001)	▼(p<0.0001)	▼ (p<0.05)
	**CRP**	NS	NS	▼ (p<0.0001)	▼(p<0.0001)	▼(p<0.0001)
	**LDL receptor**	NS	NS	NS	NS	NS
	**ACC1**	NS	NS	▼(p<0.05)	NS	▼(p<0.05)
	**ACC2**	▼ (p<0.001)	NS	NS	NS	▼(p<0.05)
	**ApoC-III**	▼(p<0.001)	NS	▼(p<0.01)	▼(p<0.0001)	NS
	**Sulf-2**	▲ (p<0.001)	NS	▼(p<0.05)	▼(p<0.001)	NS
	**PNPLA3**	▼(p<0.0001)	NS	NS	NS	NS
	**MMP-2**	▲ (p<0.01)	NS	▼(p<0.0001)	▼ (p<0.001)	NS
	**ADH-4**	NS	NS	▼(p<0.05)	▼(p<0.001)	▼(p<0.01)
	**Plasma leptin**	NS	NS	NS	NS	NS
	**Plasma CRP**	▲(p<0.001)	NS	▼(p<0.0001)	▼(p<0.0001)	NS
	**Plasma adiponectin**	▼(p<0.0001)	NS	NS	NS	▲(p<0.0001)
	**Liver triglyercide**	▲(p<0.0001)	NS	NS	NS	▼(p<0.01)
	**Liver free fatty acid**	▲(p<0.05)	NS	NS	▲(p<0.0001)	▲(p<0.0001)
	**Liver hydroxyproline**	NS	NS	NS	NS	NS
**Histological Analyses**	**NAFLD Activity Score**	▲(p<0.0001)	▼(p<0.05)	NS	▼(p<0.0001)	▼ (p<0.001)
	**Steatosis Score**	▲(p<0.0001)	NS	NS	▼(p<0.05)	NS
	**Inflammation Score**	▲(p<0.0001)	NS	NS	NS	▼(p<0.01)
	**Ballooning Score**	▲(p<0.0001)	NS	NS	▼(p<0.05)	NS
	**Fibrosis Area**	▲(p<0.001)	▼(p<0.001)	▼(p<0.001)	▼(p<0.01)	▼(p<0.0001)
	**NAFLD Activity Score**	4.8	3.4	3.6	2.5	2.6
	**Sirius red-positive area (%)**	0.84	0.56	0.53	0.59	0.48

- no significant difference

▲ significant increase

▼ significant decrease

Gemcabene also significantly reduced (low and high dose) or showed a tendency for lowering (mid-dose) the NAS compared with the vehicle-treated NASH group. Cenicriviroc, a molecule in Phase II clinical trials, which mechanism of action in NASH is targeting the suppression of CCR2/CCR5 genes, reduced NAS and fibrosis comparable to gemcabene in the STAM™ model [30].

The high-dose gemcabene group also reduced steatosis and ballooning scores. The highest dose of gemcabene showed significant improvement in the steatosis score (Fig 4) without showing a dose response, while the inflammation score showed a dose-dependent decreasing trend in the gemcabene-treated groups. These data are consistent with the gemcabene anti-inflammatory effects *in vitro*, in animal models, and in humans [13, 15, 17].

Hepatocyte ballooning is thought to be a consequence of oxidative stress-induced hepatocellular damage and is associated with the progression of NASH [39, 40]. Therefore, gemcabene may improve the NASH pathology by potentially inhibiting the oxidative consequences of inflammation.

Although gemcabene reduced histologically-assessed hepatic lipids at the highest dose level (300 mg/kg), the biochemical quantitation of liver TG was not affected at any dose. This effect is most likely attributable to (1) the excess fat in the diet and (2) the marked observed reduction of plasma TG with potential hepatic clearance perhaps due to ApoC-III mRNA and Sulf-2 mRNA reductions (see below), both of which may have obscured any gemcabene effect on liver TG levels. We rationalize the lack of effect on the hepatic lipid levels with the fact that in STAM™ mice lipid and glucose metabolism is compromised [28, 29].

Treatment with gemcabene reduced the mRNA expression levels of metabolic genes linked to lipogenesis and lipid modulation, such as ACC1, ApoC-III and ADH-4, likely causal targets for the treatment of NAFLD and targets for molecules currently in clinical development. The lipid modulator Sulf-2 gene (6-*0*-endosulfatase-2) has received poor attention in connection with NAFLD. Syndecan-1 has recently been recognized as the VLDL-remnant receptor, and Sulf-2 activity influences its sulfation status and the ability of this receptor to regulate blood triglyceride levels via VLDL remnant clearance [41]. In diabetic patients, VLDL-remnant clearance is impaired, TG levels are elevated, the number of negatively-charged sulfate groups on syndecan-1 are reduced, and Sulf-2 expression is elevated [41]. It is well known that ApoE present on VLDL-remnants, when not masked by ApoC-III, enhances VLDL-remnant clearance [42, 43]. Elevated ApoC-III levels impede VLDL remnant clearance and are associated with cardiovascular disease and NAFLD [44–47]. Mice overexpressing ApoC-III develop hypertriglyceridemia [48] and diet-induced hepatic steatosis and hepatic insulin resistance [47]. In the current study, ApoC-III mRNA levels were significantly decreased by 30% in the vehicle-treated NASH mice compared to the vehicle-treated normal mice. Gemcabene 100 and 300 mg/kg -treated groups further and significantly reduced ApoC-III mRNA expression levels, by 29% and 43%, respectively, compared to vehicle-treated NASH group. The effect of gemcabene on ApoC-III mRNA, in conjunction with its effect on Sulf-2 mRNA down-regulation, may additively or synergistically enhance VLDL remnant receptor activity and facilitate TG-rich remnant VLDL clearance. While hepatic ApoC-III mRNA level was reduced in the vehicle-treated NASH mice, a plasma TG increase was observed. However, gemcabene treatment of the NASH mice resulted in a significant and dose-dependent reduction in plasma TG. This effect may be in part related to concerted reductions of ApoC-III and Sulf-2 mRNA levels.

Although gemcabene significantly reduced the fibrosis area at the highest dose of gemcabene (300 mg/kg) (Fig 5B), a dose response was not observed. It is also established that this model induces a mild fibrosis, and hydroxyproline measurements are not very efficient at the stage of development of the disease achieved at the end of the experiment. Therefore, subsequent studies should evaluate the usefulness of gemcabene in a model displaying more advanced fibrosis.

In conclusion, gemcabene-treated STAM™ mice data from the current study infer that gemcabene may be a viable candidate for histological reduction in both NAS and fibrosis progression. Further, analysis of hepatic expression of mainly inflammation and lipid metabolism-related genes corroborate statistically meaningful changes on multiple targets associated with hepatoprotective effects on liver pathology, as it reduced the mRNA expression levels of inflammation-related genes (TNF-α, MCP-1, MIP-1β, CCR5, CCR2, NF-κB). Plasma CRP levels were also decreased by gemcabene treatment in the present study, which is in agreement with human clinical data [17], and with the downregulation of the CRP gene expression in the present study. Data from previous non-clinical and clinical studies support the gemcabene effects on the reduction in TG, VLDL clearance and reduction of ApoC-III plasma levels [13, 16].

The STAM™ model is induced with STZ, with near complete loss of pancreatic insulin production, and, therefore, translation effects of drugs on insulin sensitization are not expected. However, this model demonstrated that pleiotropic drugs such as gemcabene, and/or multi-modal combination therapy approaches, may effectively guide treatments for NASH. Therefore, additional studies in animal models with more advanced fibrosis are warranted for the elucidation of the mechanism of action of gemcabene in NAFLD/NASH. We however believe that the current nonclinical data corroborated with earlier clinical findings with gemcabene in hypercholesterolemia and hypertriglyceridemia are positive grounds for its evaluation, alone or in combination with other agents, as a therapy for NAFLD/NASH in humans.

## Supporting information

S1 File Fig A Study Plan for Assessing the Effects of Gemcabene in the STAM™ Model of NASH-HCC. Table A Information of quantitative RT-PCR. Table Ab Information of PCR primers. Table Ac Information of PCR-plate. Table B Treatment schedule. Table C Body weight and liver weight. Table D Biochemistry. Table E Gemcabene in the Landscape of the Currently Proposed Treatments for NAFLD and NASH.
(DOCX)
