# Supplementary material for: Gemcabene downregulates inflammatory, lipid-altering and cell-signaling genes in the STAM™ model of NASH
Source: PLoS One. 2018 May 30;13(5):e0194568. doi: 10.1371/journal.pone.0194568 (PMC5976190; doi:10.1371/journal.pone.0194568)
Supplement: S1 File — Fig A Study Plan for Assessing the Effects of Gemcabene in the STAM™ Model of NASH-HCC. Table A Information of quantitative RT-PCR. Table Ab Information of PCR primers. Table Ac Information of PCR-plate. Table B Treatment schedule. Table C Body weight and liver weight. Table D Biochemistry. Table E Gemcabene in the Landscape of the Currently Proposed Treatments for NAFLD and NASH. (DOCX) [file pone.0194568.s001.docx]

**S1 SUPPORTING INFORMATION**

**Gemcabene Downregulates Inflammatory, Lipid Altering and Cell-Signaling Genes in the STAM^TM^ Model of NASH**

Daniela Carmen Oniciu,^1*^, Taishi Hashiguchi^2^, Yuichiro Shibazaki ^2^, and Charles L Bisgaier^1^

^1^ Gemphire Therapeutics, Inc., 17199 N. Laurel Park, Suite 401, Livonia, MI 48152, USA

^2^ SMC Laboratories, Inc., 2-16-1 Minami-Kamata Ota-City Tokyo 144-0035 Japan

* To whom correspondence should be addressed. Email: doniciu@gemphire.com

**Contents:**

Study plan

Materials and methods

Experimental design and treatment

Results

References

**Figure Caption**:

S1 Fig A Study Plan for Assessing the Effects of Gemcabene in the STAM^TM^ Model of
NASH-HCC

**Table Legends**:

S1 Table A Information of quantitative RT-PC

S1 Table Ab Information of PCR primers

S1 Table Ac Information of PCR-plate

S1 Table B Treatment schedule

S1 Table C Body weight and liver weight

S1 Table D Biochemistry

S1 Table E Gemcabene in the Landscape of the Currently Proposed Treatments for NAFLD and NASH

**S1 Fig A Study Plan for Assessing the Effects of Gemcabene in the STAM^TM^ Model of
NASH-HCC**

# Materials and Methods

## Test substances

Gemcabene was provided by Gemphire Therapeutics Inc. It was prepared as described in [[1](#_ENREF_1)]. To prepare dosing solution, Gemcabene was weighed and dissolved with pure water as a vehicle according to the formulation instructions. Telmisartan (Micardis^®^) was purchased from Boehringer Ingelheim GmbH (Germany) and dissolved in pure water.

## Induction of NASH

NASH was induced in 40 male mice by a single subcutaneous injection of 200 µg streptozotocin (STZ, Sigma-Aldrich, USA) solution 2 days after birth and *ad libitum* feeding of a high fat diet (HFD, 57 kcal% fat, Cat# HFD32, CLEA Japan, Japan) beginning at 4 weeks of age.

## Route of drug administration

Vehicle, gemcabene, or Telmisartan were administered by oral gavage once daily, in a volume of 10 mL/kg.

## Treatment doses

Gemcabene was administered at doses of 30, 100 and 300 mg/kg/day. Telmisartan was administered at a dose of 10 mg/kg/day. The study plan for assessing the effects of gemcabene is displayed in S1 Figure A.

## Animals

C57BL/6 mice (14-day-pregnant female) were obtained from Japan SLC Inc. (Japan). Animal housing and care were in compliance with the recommendations of Directive 86/609/EEC. Animal housing and care were in compliance with the recommendations of Directive 86/609/EEC, and protocol approvals were obtained from SMC Laboratories IACUC (approval number: S062). All animals were housed and cared for in accordance with the Japanese Pharmacological Society Guidelines for Animal Use.

## Environment

The animals were maintained in a SPF facility under controlled conditions of temperature (23 ± 2°C), humidity (45 ± 10%), lighting (12-hour artificial light and dark cycles; with lights on from 8:00 to 20:00) and air exchange air exchange rate: more than 40 times/hour. A positive high pressure was maintained in the experimental room to prevent contamination of the facility.

## Animal husbandry

The animals were housed in TPX cages (CLEA Japan) with a maximum of 4 mice per cage. Sterilized Paper-Clean (Japan SLC) was used for bedding and replaced once a week.

## Food and water

Sterilized solid HFD was provided *ad libitum*, being placed in a metal lid on the top of the cage. Pure water was provided *ad libitum* from a water bottle equipped with a rubber stopper and a sipper tube. Water bottles were replaced once a week cleaned and sterilized in autoclave and reused.

## 1.9 Animal and cage identification

Mice were identified by ear punch. Each cage was labeled with a specific identification code.

## 1.10 Measurement of whole blood and plasma biochemistry

Eight-hour fasting blood samples were collected from facial vein at 3 days prior to termination.

Eight-hour fasting blood glucose was measured in whole blood using a Life Check glucose meter (EIDIA Co. Ltd., Japan). For plasma biochemistry, eight-hour fasting blood was collected in polypropylene tubes with anticoagulant (Novo-Heparin, Mochida Pharmaceutical, Japan) and centrifuged at 1,000 xg for 15 minutes at 4°C. The supernatant was collected and stored at -80°C until use. Plasma insulin levels were quantified by Ultra-Sensitive Mouse Insulin ELISA kit (Morinaga Institute of Biological Science, Inc., Japan).

On the day of termination, non-fasting blood glucose was measured in whole blood using a Life Check glucose meter. For plasma biochemistry, non-fasting blood was collected in polypropylene tubes with anticoagulant (Novo-Heparin) and centrifuged at 1,000 x g for 15 minutes at 4°C. The supernatant was collected and stored at -80°C until use. Plasma ALT, AST, ALP, GGT, BUN, creatinine, total bilirubin, triglyceride and total cholesterol levels were measured on a FUJI DRI-CHEM 7000 clinical analyzer (Fujifilm Corporation, Japan). Plasma ketone body levels were quantified with a EnzyChrom™ Ketone Body Assay Kit (BioAssay Systems, USA). Plasma ApoC-III levels was quantified with an APOC3/Apolipoprotein C III ELISA kit (LifeSpan BioSciences, In., USA). Plasma CRP levels was quantified with a Mouse High-Sensitive CRP ELISA kit (KAMIYA BIOMEDICAL COMPANY, USA). Plasma leptin and adiponectin levels were quantified with a Leptin Quantikine ELISA kit and Mouse adiponectin Quantkine ELISA kit (R&D Systems, USA), respectively.

## 1.11 Measurement of liver biochemistry

### Measurement of liver triglyceride and free fatty acid content

Liver total lipid-extracts were obtained according to the method of Folch *et al.* [[2](#_ENREF_2)]. Liver samples were homogenized in 20 volumes of chloroform-methanol (2:1, v/v) and incubated overnight at room temperature. After washing with chloroform-methanol-water (8:4:3, v/v/v), the extracts in the lower chloroform phase were evaporated to dryness, and dissolved in isopropanol. Liver triglyceride and free fatty acid contents were measured with a Triglyceride E-test and NEFA C-test, respectively (Wako Pure Chemical Industries).

### Measurement of liver hydroxyproline content

To quantify liver hydroxyproline content, frozen liver samples were processed by an alkaline-acid hydrolysis method as follows. Liver samples were defatted with 100% acetone, dried in the air, dissolved in 2N NaOH at 65°C, and autoclaved at 121°C for 20 minutes. The lysed samples (400 µL) were acid-hydrolyzed with 400 µL of 6N HCl at 121°C for 20 minutes, and neutralized with 400 µL of 4N NaOH containing 10 mg/mL activated carbon. AC buffer (2.2M acetic acid/0.48M citric acid, 400 µL) was added to the samples, followed by centrifugation to collect the supernatant. A standard curve of hydroxyproline was constructed with serial dilutions of trans-4-hydroxy-L-proline (Sigma-Aldrich) starting at 16 µg/mL. The prepared samples and standards (each 400 µL) were mixed with 400 µL chloramine T solution (Wako Pure Chemical Industries, Osaka, Japan) and incubated for 25 minutes at room temperature. The samples were then mixed with Ehrlich's solution (400 µL) and heated at 65°C for 20 minutes to develop the color. After samples were cooled on ice and centrifuged to remove precipitates, the optical density of each supernatant was measured at 560 nm. The concentrations of hydroxyproline were calculated from the hydroxyproline standard curve. Protein concentrations of liver samples were determined using a BCA protein assay kit (Thermo Fisher Scientific, USA) and used to normalize the calculated hydroxyproline values. Liver hydroxyproline levels were expressed as µg per mg protein.

## 1.12 Histological analyses

For Hematoxylin and Eosin (H&E) staining, sections were cut from paraffin blocks of liver tissue prefixed in Bouin’s solution and stained with Lillie-Mayer’s Hematoxylin (Muto Pure Chemicals Co., Ltd., Japan) and eosin solution (Wako Pure Chemical Industries). NAFLD Activity score (NAS) was calculated according to the criteria of Kleiner *et al.* [[3](#_ENREF_3)]. To visualize collagen deposition, Bouin’s fixed liver sections were stained using picro-Sirius red solution (Waldeck, Germany). For Masson Trichrome staining, the sections were stained with Masson’s Trichrome staining Kit (Sigma, USA) according to the manufacturer’s instructions.

For quantitative analysis of fibrosis area, bright field images of Sirius red-stained sections were captured around the central vein using a digital camera (DFC295; Leica, Germany) at 200-fold magnification, and the positive areas in 5 fields/section were measured using ImageJ software (National Institute of Health, USA). Samples were analyzed in a blinded fashion. The fibrosis score was determined by an average of 5 images captured at 200-fold magnification. All sections were blindly analyzed by pathologist.

## 1.13 Quantitative RT-PCR

Total RNA was extracted from liver samples using RNAiso (Takara Bio, Japan) according to the manufacturer’s instructions. One µg of RNA was reverse-transcribed using a reaction mixture containing 4.4 mM MgCl_2_ (F. Hoffmann-La Roche, Switzerland), 40 U RNase inhibitor (Toyobo, Japan), 0.5 mM dNTP (Promega, USA), 6.28 µM random hexamer (Promega), 5 x first strand buffer (Promega), 10 mM dithiothreitol (Invitrogen, USA) and 200 U MMLV-RT (Invitrogen) in a final volume of 20 µL. The reaction was carried out for 1 hour at 37°C, followed by 5 minutes at 99°C. Real-time PCR was performed using real-time PCR DICE and SYBR premix Taq (Takara Bio). To calculate the relative mRNA expression level, the expression of each gene was normalized to that of reference gene 36B4 (gene symbol: Rplp0). Information of PCR-primer sets and the plate layout was described in Tables 1S Ab and 1S Ac, respectively.

**S1 Table A Information of quantitative RT-PCR**

**S1 Table Ab Information of PCR primers**

**S1 Table Ac Information of PCR-plate**

## 1.14 Statistical tests

Statistical analyses were performed using the Bonferroni Multiple Comparison Test on GraphPad Prism 6 (GraphPad Software Inc., USA). *P* values < 0.05 were considered statistically significant. Results are expressed as mean ± SD.

# Experimental Design and Treatment

## 2.1 Study groups

S1 Table B summarizes the treatment schedule.

**Group 1: Vehicle in Normal.**  Eight normal mice were orally administered vehicle [pure water] in a volume of 10 mL/kg once daily from 6 to 9 weeks of age.

**Group 2: Vehicle in NASH.**  Eight NASH mice were orally administered vehicle in a volume of 10 mL/kg once daily from 6 to 9 weeks of age.

**Group 3: Gemcabene 30 mg/kg.** Eight NASH mice were orally administered vehicle supplemented with Gemcabene at a dose of 30 mg/kg once daily from 6 to 9 weeks of age.

**Group 4: Gemcabene 100 mg/kg.**  Eight NASH mice were orally administered vehicle supplemented with Gemcabene at a dose of 100 mg/kg once daily from 6 to 9 weeks of age.

**Group 5: Gemcabene 300 mg/kg.**  Eight NASH mice were orally administered vehicle supplemented with Gemcabene at a dose of 300 mg/kg once daily from 6 to 9 weeks of age.

**Group 6: Telmisartan 10 mg/kg.**  Eight NASH mice were orally administered pure water supplemented with Telmisartan at a dose of 10 mg/kg once daily from 6 to 9 weeks of age.

**S1 Table B Treatment schedule**

| Group | No. mice | Mice | Test substance | Dose  (mg/kg) | Volume  (mL/kg) | Regimen | Sacrifice  (wks) |
| --- | --- | --- | --- | --- | --- | --- | --- |
| 1 | 8 | Normal | Vehicle | - | 10 | PO, QD,  6 wks - 9 wks | 9 |
| 2 | 8 | STAM | Vehicle | - | 10 | PO, QD,  6 wks - 9 wks | 9 |
| 3 | 8 | STAM | Gemcabene | 30 | 10 | PO, QD,  6 wks - 9 wks | 9 |
| 4 | 8 | STAM | Gemcabene | 100 | 10 | PO, QD,  6 wks - 9 wks | 9 |
| 5 | 8 | STAM | Gemcabene | 300 | 10 | PO, QD,  6 wks - 9 wks | 9 |
| 6 | 8 | STAM | Telmisartan | 10 | 10 | PO, QD,  6 wks - 9 wks | 9 |

## 2.2 Animal monitoring and sacrifice

The viability, clinical signs and behavior were monitored daily. Body weight was recorded before the treatment. Mice were observed for significant clinical signs of toxicity, moribundity and mortality approximately 60 minutes after each administration. The animals were sacrificed by exsanguinations through direct cardiac puncture under isoflurane anesthesia at 9 weeks of age.

# Results

## 3.1 Body weight changes and general condition (S1 Table C)

Mean body weight of the Vehicle-treated NASH mice was significantly lower than that of the Vehicle-treated Normal mice during the treatment period. Mean body weight of the Telmisartan-treated mice was significantly lower than that of the Vehicle-treated NASH mice from Day 10 to Day 21. There were no significant changes in mean body weight during the treatment period between the Vehicle-treated NASH mice and the Gemcabene treatment groups. During the treatment period, mice found dead before reaching Day 21 was as follows; One out of 8 mice was found dead in the Telmisartan-treatment group.

## 3.2 Body weight on the day of termination

The Vehicle-treated NASH mice showed a significant decrease in mean body weight on the day of termination compared with the Vehicle-treated Normal mice. The Telmisartan-treated mice showed a significant decrease in mean body weight on the day of termination compared with the Vehicle-treated NASH mice. There were no significant differences in mean body weight on the day of termination between the Vehicle-treated NASH mice and the Gemcabene-treated groups.

## 3.3 Liver weight and liver-to-body weight ratio (S1 Table C)

The Vehicle-treated NASH mice showed a significant increase in mean liver weight compared with the Vehicle-treated Normal mice. The Gemcabene-treated 100 and 300 mg/kg mice showed significant increases in mean liver weight compared with the Vehicle-treated NASH mice. The Telmisartan-treated mice showed a significant decrease in mean liver weight compared with the Vehicle-treated NASH mice. There was no significant difference in mean liver weight between the Vehicle-treated NASH mice and the Gemcabene-treated 30 mg/kg mice.

The Vehicle-treated NASH mice showed a significant increase in mean liver-to-body weight ratio compared with the Vehicle-treated Normal mice. The Gemcabene-treated 100 and 300 mg/kg mice showed significant increases in mean liver-to-body weight ratio compared with the Vehicle-treated NASH mice. There were no significant differences in mean liver-to-body weight ratio between the Vehicle-treated NASH group and any of the other treatment groups.

**S1 Table C Body weight and liver weight**

## 3.4 Biochemistry

Biochemistry results are summarized in S1 Table D.

### 3.4.1 Blood analysis 3 days prior to termination after 8 hours of fasting

Fasting whole blood glucose: The Vehicle-treated NASH mice showed a significant increase in fasting whole blood glucose levels compared with the Vehicle-treated Normal group. The Telmisartan-treated mice showed a significant increase in fasting whole blood glucose levels compared with the Vehicle-treated NASH mice. There were no significant differences in fasting whole blood glucose levels between the Vehicle-treated NASH mice and the Gemcabene-treated mice.

Fasting plasma insulin: The Vehicle-treated NASH mice showed a significant decrease in fasting plasma insulin levels compared with the Vehicle-treated Normal mice. There were no significant differences in fasting plasma insulin levels between the Vehicle-treated NASH mice and any of the other treatment groups.

### 3.4.2 Blood analysis at termination (S1 Table D)

Whole blood glucose: The Vehicle-treated NASH mice showed a significant increase in whole blood glucose levels compared with the Vehicle-treated Normal mice. The Telmisartan-treated mice showed a significant increase in whole blood glucose levels compared with the Vehicle-treated NASH mice. There were no significant differences in whole blood glucose levels between the Vehicle-treated NASH mice and the Gemcabene-treated mice.

### 3.4.3 Plasma ALT (S1 Table D)

The Vehicle-treated NASH mice showed a significant increase in plasma ALT levels compared with the Vehicle-treated Normal mice. The Gemcabene-treated 100 mg/kg mice showed a significant decrease in plasma ALT levels compared with the Vehicle-treated NASH mice. There were no significant differences in plasma ALT levels between the Vehicle-treated NASH mice and any of the other treatment groups.

### 3.4.4 Plasma AST (S1 Table D)

There were no significant differences in plasma AST levels between the Vehicle-treated NASH mice and any of the treatment groups.

### 3.4.5 Plasma ALP (S1 Table D)

The Gemcabene-treated 100 and 300 mg/kg mice and Telmisartan-treated mice showed significant increases in plasma ALP levels compared with the Vehicle-treated NASH group. There were no significant differences in plasma ALP levels between the Vehicle-treated NASH mice and any of the other treatment groups.

### 3.4.6 Plasma GGT (S1 Table D)

There were no significant differences in plasma GGT levels between the Vehicle-treated NASH mice and any of the treatment groups.

### 3.4.7 Plasma BUN (S1 Table D)

The Telmisartan-treated mice showed a significant increase in plasma BUN levels compared with the Vehicle-treated NASH mice. There were no significant differences in plasma BUN levels between the Vehicle-treated NASH mice and any of the other treatment groups.

### 3.4.8 Plasma creatinine (S1 Table D)

The Vehicle-treated NASH mice showed a significant decrease in plasma creatinine levels compared with the Vehicle-treated Normal mice. The Gemcabene-treated 300 mg/kg mice showed a significant increase in plasma creatinine levels compared with the Vehicle-treated NASH group. There were no significant differences in plasma creatinine levels between the Vehicle-treated NASH mice and any of the other treatment groups.

### 3.4.9 Plasma total bilirubin (S1 Table D)

There were no significant differences in plasma total bilirubin levels between the Vehicle-treated NASH mice and any of the treatment groups.

### 3.4.10 Plasma ketone body (S1 Table D)

The Vehicle-treated NASH mice showed a significant increase in plasma ketone body levels compared with the Vehicle-treated Normal mice. There were no significant differences in plasma ketone body levels between the Vehicle-treated NASH mice and any of the other treatment groups.

### 3.4.11 Liver triglyceride (S1 Table D)

The Vehicle-treated NASH mice showed a significant increase in liver triglyceride contents compared with the Vehicle-treated Normal mice. The Telmisartan-treated mice showed a significant decrease in liver triglyceride content compared with the Vehicle-treated NASH mice. There were no significant differences in liver triglyceride content between the Vehicle-treated NASH mice and Gemcabene-treated groups.

### 3.4.12 Liver hydroxyproline (S1 Table D)

There were no significant differences in liver hydroxyproline contents between the Vehicle-treated NASH mice and any of the treatment groups.

### 3.4.13 Plasma triglyceride (S1 Table D)

The Vehicle-treated NASH mice showed a significant increase in plasma triglyceride levels compared with the Vehicle-treated Normal mice. The Gemcabene-treated mice showed significant decreases in plasma triglyceride levels compared with the Vehicle-treated NASH mice in a dose-dependent manner. There was no significant difference in plasma triglyceride levels between the Vehicle-treated NASH mice and the Telmisartan-treated mice.

### 3.4.14 Plasma total cholesterol (S1 Table D)

The Vehicle-treated NASH mice showed a significant increase in plasma total cholesterol levels compared with the Vehicle-treated Normal mice. The Gemcabene-treated 100 and 300 mg/kg mice and the Telmisartan-treated mice showed significant increases in plasma total cholesterol levels compared with the Vehicle-treated NASH mice. There was no significant difference in plasma total cholesterol levels between the Vehicle-treated NASH mice and the Gemcabene-treated 30 mg/kg mice.

# REFERENCES

1. Ando HY, Butler DE, Dozeman GJ, Calcium dicarboxylate ethers, methods of making same, and treatment of vascular disease and diabetes therewith United States patent US 7,141,608. 2006 Sep. 10, 2004.

2. Folch J, Lees M, Sloane Stanley GH. A simple method for the isolation and purification of total lipides from animal tissues. J Biol Chem. 1957;226(1):497-509. PubMed PMID: 13428781.

3. Kleiner DE, Brunt EM, Van Natta M, Behling C, Contos MJ, Cummings OW, et al. Design and validation of a histological scoring system for nonalcoholic fatty liver disease. Hepatology. 2005;41(6):1313-21. doi: 10.1002/hep.20701. PubMed PMID: 15915461.

4. Leclercq IA, Lebrun VA, Starkel P, Horsmans YJ. Intrahepatic insulin resistance in a murine model of steatohepatitis: effect of PPARgamma agonist pioglitazone. Lab Invest. 2007;87(1):56-65. doi: 10.1038/labinvest.3700489. PubMed PMID: 17075577.

5. Kawai D, Takaki A, Nakatsuka A, Wada J, Tamaki N, Yasunaka T, et al. Hydrogen-rich water prevents progression of nonalcoholic steatohepatitis and accompanying hepatocarcinogenesis in mice. Hepatology. 2012;56(3):912-21. doi: 10.1002/hep.25782. PubMed PMID: 22505328.

6. Ji HF, Shen L. On the mechanism of action of vitamin E for nonalcoholic steatohepatitis. Hepatology. 2011;53(3):1067. doi: 10.1002/hep.23950. PubMed PMID: 20890896.

7. Xu R, Tao A, Zhang S, Deng Y, Chen G. Association between vitamin E and non-alcoholic

steatohepatitis: a meta-analysis. (3) ed. Int J Clin Exp Med2015. p. 3924-34.

8. Suzuki-Kemuriyama N, Matsuzaka T, Kuba M, Ohno H, Han SI, Takeuchi Y, et al. Different Effects of Eicosapentaenoic and Docosahexaenoic Acids on Atherogenic High-Fat Diet-Induced Non-Alcoholic Fatty Liver Disease in Mice. PLoS One. 2016;11(6):e0157580. doi: 10.1371/journal.pone.0157580. PubMed PMID: 27333187; PubMed Central PMCID: PMCPMC4917109.

9. Verbeke L, Farre R, Trebicka J, Komuta M, Roskams T, Klein S, et al. Obeticholic acid, a farnesoid X receptor agonist, improves portal hypertension by two distinct pathways in cirrhotic rats. Hepatology. 2014;59(6):2286-98. doi: 10.1002/hep.26939. PubMed PMID: 24259407.

10. Lefebvre. E, Hashiguchi. T, H. Jenkins, Yoneyama. H, Friedman. SL, Wolfgang. GHI. Anti-Fibrotic and Anti-Inflammatory Activity of the Dual CCR2 and CCR5 Antagonist Cenicriviroc in a Mouse Model of NASH. The Liver Meeting AASLD, 2013 Abstract 30.

11. Staels B, Rubenstrunk A, Noel B, Rigou G, Delataille P, Millatt LJ, et al. Hepatoprotective effects of the dual peroxisome proliferator-activated receptor alpha/delta agonist, GFT505, in rodent models of nonalcoholic fatty liver disease/nonalcoholic steatohepatitis. Hepatology. 2013;58(6):1941-52. doi: 10.1002/hep.26461. PubMed PMID: 23703580.

12. Klein T, Fujii M, Sandel J, Shibazaki Y, Wakamatsu K, Mark M, et al. Linagliptin alleviates hepatic steatosis and inflammation in a mouse model of non-alcoholic steatohepatitis. Med Mol Morphol. 2014;47(3):137-49. doi: 10.1007/s00795-013-0053-9. PubMed PMID: 24048504.

13. Harriman G, Greenwood J, Bhat S, Huang X, Wang R, Paul D, et al. Acetyl-CoA carboxylase inhibition by ND-630 reduces hepatic steatosis, improves insulin sensitivity, and modulates dyslipidemia in rats. Proc Natl Acad Sci U S A. 2016;113(13):E1796-805. doi: 10.1073/pnas.1520686113. PubMed PMID: 26976583; PubMed Central PMCID: PMCPMC4822632.

14. Chen Z, Vigueira PA, Chambers KT, Hall AM, Mitra MS, Qi N, et al. Insulin resistance and metabolic derangements in obese mice are ameliorated by a novel peroxisome proliferator-activated receptor gamma-sparing thiazolidinedione. J Biol Chem. 2012;287(28):23537-48. doi: 10.1074/jbc.M112.363960. PubMed PMID: 22621923; PubMed Central PMCID: PMCPMC3390629.

15. Erstad DJ FC, Masia R, Ferreira DDS, Wei L, Choi J-K,. A novel farnesoid X receptor agonist: EDP-305, reduces fibrosis progression in animal models of fibrosis Journal of Hepatology , Volume 66 , Issue 1 , S165.

16. Krupinski J, Morgan N, Kozhich A, Manoj C, Morin P, Christian R. Effects of BMS-986036 (Pegulated Growth Factor 21) on Hepatic Steatosis and Fibrosis in a Mouse Model of Nonalcoholic Steatohepatitis. NASH-TAG Conference 2017, Abstract Boob. 2017. p. 15.

**S1 Table D Biochemistry**

**S1 Table E Gemcabene in the Landscape of the Currently Proposed Treatments for NAFLD and NASH**

| **Drug / Drug Candidate** | **Nonclinical Animal Model** | **Nonclinical Observations** | **Clinical Study** | **Clinical Primary End-Point** | **Clinical Results and Notes** |
| --- | --- | --- | --- | --- | --- |
| **Pioglitazone**  PPAR-γ agonist  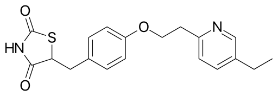 | MCD [[4](#_ENREF_4)]  STAM [[5](#_ENREF_5)] | ↓TGF- β, ↓serum ALT, ↓total hepatic lipids, ↓inflammation area, ↓hepatocyte ballooning | **PIVENS**  NIDDK  96 weeks  NCT00063622  Phase 3 study | ↓by ≥1pt in ballooning **AND** no ↑ in ﬁbrosis^a, b^ **AND** *either* a ↓in NAS to ≤3 *or* a  ↓in NAS by ≥2pt with ≥1pt ↓in either lobular inflammation or steatosis | Pioglitazone 30 mg daily 34% (NS) vs Placebo 19%  **Note**: used off label  Ph 2 clinical study at Kaohsiung Medical University (Taiwan) |
| **Vitamin E**  Oxidative stress reduction  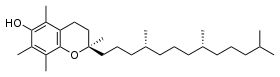 | N/A | Presumably antioxidant properties of all isomers [[6](#_ENREF_6)] | **PIVENS**  NIDDK  96 weeks  NCT00063622  (see also [[7](#_ENREF_7)])  Phase 3 study | ↓by ≥1pt in ballooning AND no ↑ in ﬁbrosis^a^ AND either a ↓in NAS to ≤3 or a  ↓in NAS by ≥2pt with ≥1pt ↓in either lobular inflammation or steatosis. | Vitamin E 800 IU daily (Nature Made brand) 43% (p=0.001) vs Placebo 19%  **Note**: administered off-label to adults  A Phase 2 study in China (Zhejiand Med Company) ongoing |
| **EPA-E**  Potential Diacylglycerol O-Acyltransferase inhibitor (DGAT1 and DGAT2)  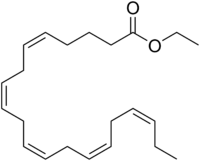 | Atherogenic high-fat fed (AHF) mice [[8](#_ENREF_8)] | ↓hepatic lobular inflammation and ↑serum transaminase; ↓hepatic triacylglycerol (TG) | **EPA-E**  Mochida Pharmaceuticals Inc.  52 weeks  NCT01154985  Phase 2 study | ↓in NAS to ≤3 *or* a ↓in NAS by ≥2pt with contribution from more than one domain **AND** no ↑ in ﬁbrosis^a^ | Placebo 40%  EPA-E 1800 mg daily 37% (NS)  EPA-E 2700 mg daily 35.9% (NS)  **Note**: administered off-label in adults, not recommended in children |
| **Obeticholic Acid (OCA)**  FXR agonist (semi-synthetic bile acid)  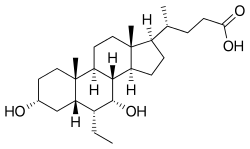 | Thioacetamide (TAA)-intoxicated and bile-duct–  ligated (BDL) rats [[9](#_ENREF_9)] | ↓FXR expression  ↓portal pressure  ↓intrahepatic vascular resistance  increased eNOS activity | **FLINT**^b^  Intercept Pharmaceuticals  72 weeks  NCT01265498  Phase 2 study  **[REGENERATE]**  ~6 years, first readout 18 months  10 mg and 25 mg OCA qd  NCT02548351  Phase 3 study | ↓ by ≥2pt in NAS **AND** no ↑ in ﬁbrosis^a^ | FLINT Study: 46% of patients on OCA achieved decrease ≥ 2 points with no worsening of fibrosis in NAS) vs. 21% for placebo (P<0.001). 35% on OCA had reduction in fibrosis vs. 19% on placebo (P=0.01).  **Note**: The US FDA issues a safety communication warning for serious liver injury and death associated with incorrect dosing of Intercept’s Ocaliva (Sept. 2017) |
| **Cenicriviroc (CVC)**  Dual CCR2-CCR5 Antagonist  *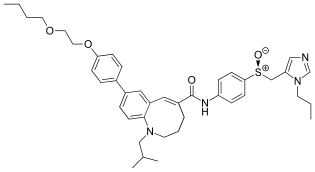* | STAM [[10](#_ENREF_10)] | ↓fibrosis - trend towards a dose response effect  no downregulation of TNF-α, MCP-1, and TIMP-1 mRNA expression levels | **CENTAUR**  Tobira Therapeutics, Inc  104 weeks^c^  NCT02217475  Phase 2 study  **Currently in Phase 3** | ↓in NAS by ≥2pt with contribution from more than one domain **AND** no ↑ in fibrosis^d^ | CVC did not meet the primary endpoint of 2 pt reduction in NAS vs. placebo  CVC reduced fibrosis by at least 1 stage without worsening of NASH vs. placebo (20% vs. 13%).  CVC improved fibrosis by ≥1 stage in 35% of patients vs. 20% for placebo. |
| **Elafibranor (GFT505)**  PPAR-α/δ agonist  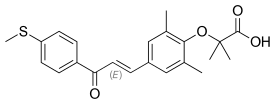 | Western diet [WD]-fed human apolipoprotein E2 [hApoE2] transgenic mice  methionine- and choline-deficient diet-fed db/db mice  CCl4 -induced fibrosis in rats [[11](#_ENREF_11)]  hApoE2 knock-in/PPAR-α knockout mice | liver-protective effects on steatosis, inflammation, and fibrosis  liver dysfunction markers  ↓hepatic lipid accumulation  inhibited IL-1β, TNFα, F4/80  ↓MMP 2, ↓COL2A1 ↓COL1A1 ↓COL1A2 mRNA expression levels  PPAR-α-independent mechanisms | **GOLDEN**  Genfit  52 weeks  NCT01694849  Phase 2 study  **[RESOLVE-IT]**  double-blind, placebo-controlled (2:1, 120 mg GFT505 or placebo once daily), conducted in ~ 2 000 NASH patients with F2 or F3 fibrosis, at 250 centers worldwide in patients (NAS≥4)  Phase 3 study | Reduction to zero in any one of ballooning, lobular inflammation or steatosis with no worsening in fibrosis^a^  Resolution defined by a score of B_0_ and lobular inflammation score of 0/1 without worsening of fibrosis^a^ | GFT505 80 mg daily23%  GFT505 120 mg daily 21%  Post-hoc analysis^e^: 120-mg GFT505 vs placebo:19% vs 12%; odds ratio = 2.31; 95% confidence interval: 1.02-5.24; P = .045 (80 mg vs placebo 19% vs 12%) serum  ↓α2-macroglobulin, haptoglobin, TIMP1 |
| **Linagliptin**  Dipeptidyl peptidase (DPP)-4 inhibitor  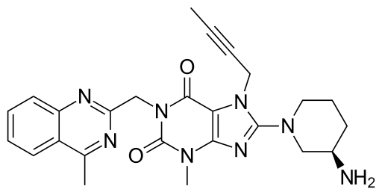 | STAM [[12](#_ENREF_12)] | ↓NAS score from 4.1 ± 0.4 to 2.4 ± 0.4 in 2 weeks (Telmisartan 1.4 ± 0.3)  ↓ SOCS-3, IFN-γ, TNF-α mRNA levels  distribution of radiolabeled linagliptin was heterogeneous with the highest density associated with interlobular bile ducts and portal tracts | Boehringer Ingelheim  NCT01084005  Phase 3 study  **Currently in Phase 4**  (approved treatment for T2DM) | HbA1c Change from baseline to Week 24 | Linagliptin provided meaningful placebo-adjusted HbA1c reductions of -0.68% (95% confidence interval: -1.19, -0.17), -1.08% (-2.02, -0.14) and -0.62% (-1.25, 0.01) after 24, 18 and 12 weeks, respectively. |
| **NDI-010976 (GS-0976)**  Allosteric ACC1 and ACC2 inhibitor   | Zucker diabetic fatty rats [[13](#_ENREF_13)] | ↓steatosis, ↑glucose-stimulated insulin secretion, ↓reduces hemoglobin A1c | Gilead/Nimbus  NCT02876796  Phase 1 study  **Currently in Phase 2** | Pharmacodynamic (PD) effects of GS-0976 (NDI-010976) on fractional de novo lipogenesis (DNL) following a single oral dose administration in overweight and/or obese, but otherwise healthy, male adults. | Statistically significant improvements in liver fat content and noninvasive markers of fibrosis, via inhibition of hepatic de novo lipogenesis (DNL) |
| **MSDC0602K**  PPARγ agonist  **** | DIO mice  PPARγ -/- [[14](#_ENREF_14)] | ↑insulin sensitivity, ↓adipose tissue inflammation,  ↓hepatic lipogenesis and gluconeogenesis: PPARγ was not required to suppress these pathways. | **EMMINENCE**  Octeta Pharmaceuticals and Covance  NCT02784444  Phase 2 study | Hepatic histological improvement in NASH defined as a decrease in the NAS (NASH CRN scoring) by at least 2 points with no concurrent worsening of fibrosis at 12 months | In progress |
| **EDP-305**  FXR Agonis**t**  (non-bile acid with steroid and non-steroid components and no carboxylic acid group) | CDA, HFD and BDL rodent models of NASH Bile Duct Ligated Rats  in vitro [[15](#_ENREF_15)]  STAM [[15](#_ENREF_15)] | ↓liver fibrosis  ↑LDLr  ↓key genes involved in inflammation and fibrosis  ↓NAS | Enanta Pharmaceuticals  NCT02918929  Phase 1 study  **Currently in Phase 2** | Safety data in NASH | Safety and PK |
| **BMS 986036 (Pegylated Fibroblast Growth Factor 21 FGF21)**  Fibroblast growth factor 21 (FGF21) is a novel hormone-like polypeptide | STAM [[16](#_ENREF_16)] | ↑serum adiponectin; ↓steatosis, ↓NAS ↓fibrosis statistically significant | BMS  NCT02413372  Phase 2 study | Change in percent hepatic fat fraction (%) by Magnetic Resonance Imaging (MRI) | In progress  **Note:** When administered exogenously, BMS 986036 has been shown to have beneficial effects on food intake, body weight, and metabolism |
| **Liraglutide (Victoza)**  Long-acting glucagon-like peptide-1 (GLP-1) receptor agonist  C_172_H_265_N_43_O_51_ 3751.202 g/mol | CCAAT/enhancer-binding protein (C/EBP) homologous protein (CHOP) KO mice fed a diet high in fat, fructose, and cholesterol (HFCD) [[16](#_ENREF_16)]. | ↓steatohepatitis, ↑insulin sensitivity, ↓de novo lipogenesis, ↓endoplasmic reticulus stress-mediated hepatocyte apoptosis | **LEAN**  Novo Nordisk  48 weeks  NCT01237119  Phase 2 study  **Currently in Phase 3**  (approved treatment for T2DM) | Resolution of NASH without worsening of fibrosis^a^ | Placebo 9%  Liraglutide (Victoza) 1.8 mg once daily, subcutaneous injection 39% (p=0.02)  **Note**: Liraglutide binds to the same receptors as does the endogenous metabolic GLP-1 hormone that stimulates insulin secretion |
| **Gemcabene**  **** | STAM | ↓TNF-α, MCP-1, MIP-1β, CCR5, CCR2, NF-κB,  TIMP-1, MMP-2, ACC1, ApoC-III, sulf-2  ↓NAS ↓fibrosis | Gemphire Therapeutics  Planning Phase 2 study  **Currently in Phase 2b** (dyslipidemia) |  | **Note**: in dyslipidemia patients reduction of plasma VLDL-C, LDL-C, TGs, ApoB and elevation of HDL-C |

a Worsening in fibrosis was defined by an increase in one stage of fibrosis as per the Kleiner fibrosis classification [[3](#_ENREF_3)].

b This did not include any stipulation about absence of worsening in fibrosis. Resolution defined as either not NAFLD, or NAFLD but not NASH.

c Primary end-point assessed at 52 weeks: pt, point, NS, not significant.

d Worsening in fibrosis defined as either progression to stage 3 or stage 4 (if stage 3 at baseline) in the Kleiner fibrosis classification [[3](#_ENREF_3)].

e This represented a *post hoc* analysis of a modified definition of the primary end-point. Improvement in NASH histological activity (both lobular inflammation and ballooning) highly correlates with fibrosis regression.
